# Parents’ Perception of Family-Centered Music Therapy with Stable Preterm Infants

**DOI:** 10.3390/ijerph182312813

**Published:** 2021-12-05

**Authors:** Susann Kobus, Marlis Diezel, Britta Huening, Monia Vanessa Dewan, Ursula Felderhoff-Mueser, Nora Bruns

**Affiliations:** Clinic for Pediatrics I, Essen University Hospital, University of Duisburg-Essen, 45147 Essen, Germany; marlis.diezel@gmail.com (M.D.); Britta.Huening@uk-essen.de (B.H.); Monia.Dewan@uk-essen.de (M.V.D.); Ursula.Felderhoff@uk-essen.de (U.F.-M.); nora.bruns@uk-essen.de (N.B.)

**Keywords:** music therapy, preterm infants, premature infants, neonatal intensive care unit, parents, family, family-centered

## Abstract

Premature birth places considerable demands on preterm infants and their families. Most of these infants are treated on a neonatal intensive care unit immediately after birth, leading to psychosocial stress for parents and making it more difficult to build a stable parent-child bond. We hypothesized that accompaniment with live music therapy by a music therapist supports the parents to get in contact with their child and to promote the parents’ wellbeing. Preterm infants born at less than 32 gestational weeks received creative music therapy twice a week until discharge. At the time of discharge, the parents were asked to complete a Likert-style questionnaire to evaluate the music therapy. Six items related to socio-demographic characteristics, 4 items to observations on the infant and 10 items to personal perception. Of 40 preterm infants receiving music therapy, 32 (80%) parents completed the questionnaires. Thirty (94%) of these parents were able to relax during the music therapy session. Relaxation in their infants was observed by 29 (91%) during and by 28 (88%) after music therapy. Parents perceived music therapy as a positive change and enrichment during their infant’s hospital stay. All parents were thankful for the music therapy they received. Music therapy supports the parents of preterm infants in the first time after birth until discharge from the hospital.

## 1. Introduction

Premature infants are children born before the end the 37th week of pregnancy. Every year, about 15 million children around the world are born prematurely and the trend is increasing [1]. Although premature infants are not primarily ill, organ immaturity can lead to various medical problems in the short and long term. In recent years, the neurodevelopmental outcomes of former premature infants have greatly improved thanks to advances in perinatal care, including prenatal steroids, surfactant replacement therapy, non-invasive ventilation, nutritional therapy, and an increase in active treatment [2].

The care for premature infants has changed considerably in the last few decades, not only due to medical advances, but also as a result of expanded psychosocial care. With the decision of the Federal Joint Committee (G-BA), the decision-making body of the joint self-administration in the german health care system, the politicians recognized the need for family-centered care [3]. In the perinatal center of the University Hospital Essen, family-centered care has been established since 2007 by the parent advisory service "Frühstart". The parents of premature infants and ill newborns are cared for from high-risk pregnancy to home. Since 2016, creative, family-centered music therapy has also been offered as part of family-centered care.

Music therapy serves as a means of interaction, as a non-verbal form of communication and to promote the child’s individual development [4]. In addition, it actively strengthens parents in looking after their children [5]. A qualitative study with premature infants born before 32 weeks’ gestation showed that creative music therapy can support the parent-child relationship. The musical interaction evoked feelings of joy and relaxation in the parents and encouraged them to interact more deeply with their child. In premature infants, music therapy exerts a stabilizing and relaxing effect with respect to the general behavior, physiological parameters, and sleep patterns [6,7,8,9]. Some of these positive effects can already be observed during the intervention [10]. Live music therapy has a stabilizing effect on vital functions and is also effective during sleep [11]. It has recently been shown that live music therapy enhances the functional brain activity and even the brain structure in premature infants [12].

Not only the premature infant itself, but also its parents are exposed to-mainly emotional-stress. The normal establishment of a parent-child bond is made more difficult by the fact that the child is first given medical care after birth and the parents see it connected to cables at first sight. In addition to the external framework conditions of a neonatal intensive care unit, parental fears make it more difficult to build bonds between parents and children and can have a negative impact on the development of the preterm infant [13,14]. Nevertheless, parents play a central role in promoting the development of premature infants. Their inclusion in the care is now considered a necessary part of the treatment of premature babies [15].

Based on the previous findings in the field of family-centered work with premature infants in an inpatient setting and the research results in the area of family-centered music therapy, we examined how parents perceived the in-patient family-centered music therapy care of their premature infants, born before 32 weeks’ gestation.

## 2. Materials and Methods

### 2.1. Study Design

The results of the parent survey presented here were collected as part of a prospective, randomized controlled clinical trial (German Clinical Trials Registry number: DRKS00025753) on music therapy in 80 premature infants born before 32 gestational weeks. The infants were randomly assigned to either music therapy group or no music therapy group. Apart from receiving music therapy, medical care did not differ between the therapy and the control group. Parents whose infant was randomized into the intervention group received the questionnaires evaluated in this study before discharge.

### 2.2. Eligibility and Recruitment

Preterm infants born with a gestational age with less than 32 weeks could be included in the study. The infants were born at the University Hospital Essen between October 2018 and May 2021. With a minimum age of three days, parental declaration of consent was obtained during the first week of life. Exclusion criteria were congenital hearing disorders, periventricular haemorrhagic infarction, cerebral malformations, and underlying diseases that impair neurological development.

During the first week of life, parents were approached and infants included in the study after the parents signed informed consent. The study was approved by the local ethics committee of the Medical Faculty of the University of Duisburg-Essen (18-8035-BO).

### 2.3. Randomization

Prior to the start of the study, block randomization (20 participants per block, allocation 1:1) was performed and opaque sealed envelopes containing the randomization group were prepared by NB. After inclusion in the study, a consecutive study number was assigned to each patient and an envelope with the corresponding number was unsealed by the person who had recruited the infant (MD, MVD, NB or SK).

### 2.4. Intervention

A music therapy session was carried out by a qualified music therapist twice a week in all clinically stable patients in the intervention group from the second week of life until discharge. The music therapist is a trained music therapist with a university degree in music (Master of Arts) and qualified as a specialist in music therapy in neonatology. As a specialist in music therapy in neonatology she regularly gives educational trainings.

The timing of the individual therapy session was agreed between the music therapist, the nursing staff and the parents. During therapy, the child was in the same position as before therapy, e.g., the incubator, heated cod or parent’s arm or breast during kangaroo care. The parents could be present during the intervention; this was explicitly requested, but not a condition. The music therapist sang melodies with a few musical notes or played the sansula in order to make the sessions comparable. Depending on the infant’s condition, the singing was adapted and individual melodies were played on the sansula. The tempo was adjusted according to the breathing and the heart rates.

The sansula has a room-filling, long-lasting and soft sound. The instrument consists of a wooden ring covered with a drum skin, on which a small kalimba is attached. The sound and the vibrations are transmitted through the drum skin. If the sansula is held in direction of the child while plucking the sound tongues, a feeling of being enveloped in the sound can arise. The music therapy was prepared and carried out individual for each child, not in the entire intensive care room.

Detailed information on each music therapy session including the duration, presence of parent/guardian, the instrument/singing, pre- and post-therapy infants’ behavioral states and vital signs were documented in a form.

### 2.5. Questionnaires

At the time of discharge, the parents filled out an anonymous Likert-style questionnaire on how they had perceived music therapy. The questionnaire consisted of three parts. Six items related to socio-demographic characteristics of the parents, four items to observations on the infant from the parents and 10 items to personal perceptions of the parents. The parents put the completed questionnaire in the mailbox of the parent advisory service on the neonatal ward. It took about five minutes to fill out the questionnaire. The first part of the questionnaire contained fourteen questions about music therapy during the inpatient stay. Sociodemographic characteristics were recorded in six further questions.

### 2.6. Statistical Analysis

The socio-demographic characteristics of the parents of the therapy group were given as absolute frequencies and percentages, and the variables of the written survey in percent. All graphics were created with Microsoft Excel.

## 3. Results

### 3.1. Patients

During the study period, 144 premature infants were born before 32 weeks’ gestation at the University Hospital Essen, Germany. Eighty premature infants were included in the study, 40 in the therapy group and 40 in the control group. Clinical data of the patients included in the therapy and control groups are shown in Table 1. 64 premature infants were not included. The reasons for not including were birth in another hospital (*n* = 3, 5%), death before recruitment (*n* = 11, 17%), transfer to another hospital (*n* = 4; 6.0%), cerebral hemorrhage ° III (*n* = 3; 5%), critical illness or death of the mother (*n* = 2; 3%), insufficient knowledge of German to understand the study objective (*n* = 3; 5%), inpatient stay during the study stop within the corona pandemic (*n* = 6; 9%) and a lack of interest in participating (*n* = 32; 50%).

### 3.2. Music Therapy Sessions

605 music therapy sessions were conducted in the therapy group between corrected gestational ages of 24 + 5 and 43 + 5 weeks. Parents were present in 252 (42%) sessions. The mean duration of each music therapy session was 24.2 ± 8.6 min (range 10 to 50 min).

### 3.3. Questionnaires

We received 32 completed questionnaires from the parents of the 40 children in the therapy group. Three children died during their hospital stay and no questionnaire was filled out by parents of five other children. Sociodemographic characteristics of the parents who completed a questionnaire are shown in Table 2.

**Figure 1:**
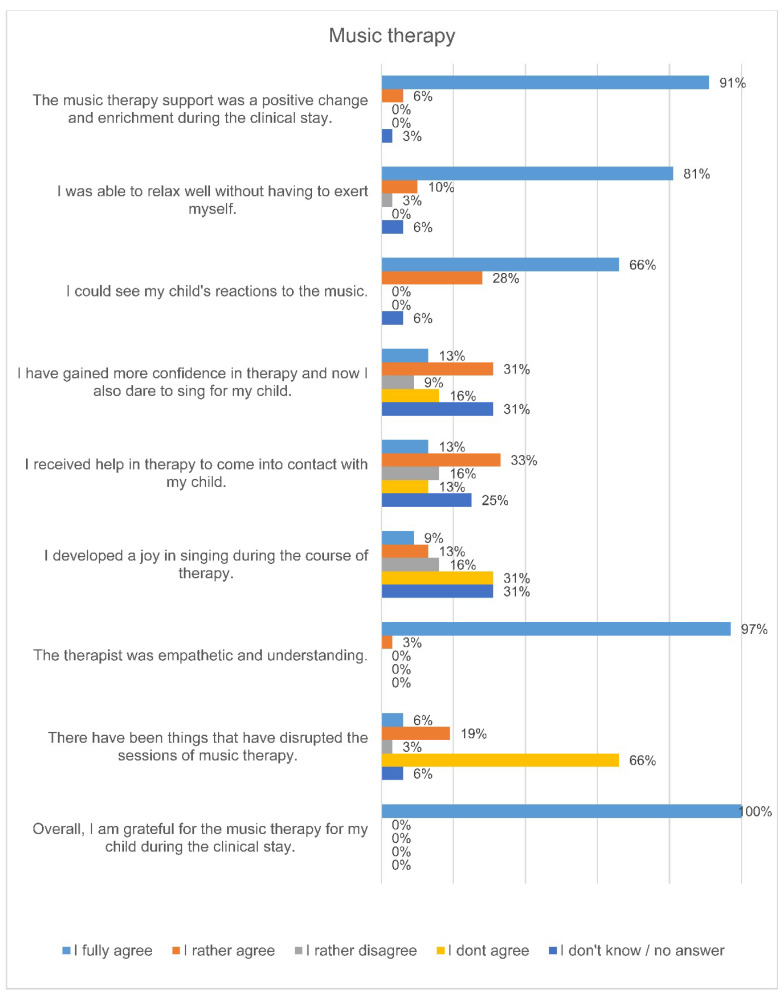
Parents’ perceptions during inpatient music therapy care of their premature infant.

The answers of the questionnaires showed that the parents perceived the music therapy support positively (Figure 1). 100% of the responding parents said that they were grateful for the music therapy they received. 94% of the parents at the University Medicine Essen stated that they could observe their child’s reactions to the music during the music therapy interventions. These included smiling, grimacing, blinking, making little voices, pacifying, lounging and deep breaths.

**Figure 2:**
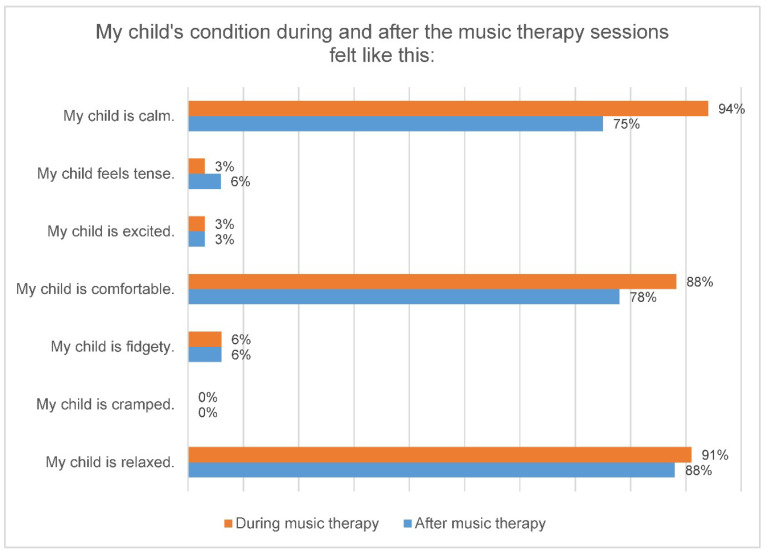
Parents’ perception of the condition of their premature infant during and after a music therapy session.

During music therapy, 94% (*n* = 30) of the parents found their child calm, in 75% (*n* = 24) this continued even after music therapy. Likewise, 91% (*n* = 29) of the children looked relaxed for their parents during music therapy and 88% (*n* = 28) of the children also after music therapy (Figure 2). In our study, 88% (*n* = 28) of the parents stated that their child felt comfortable during the music therapy and 78% (*n* = 25) of the parents were able to observe this even after the music therapy. Overall, 94% (*n* = 30) of the parents said that the music therapy was good for their child and 78% (*n* = 25) for them too.

**Figure 3:**
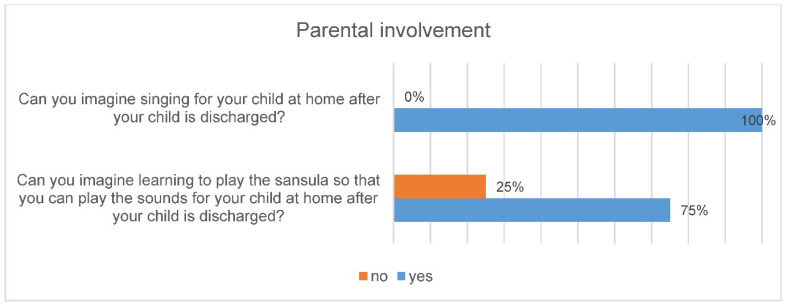
Involvement of parents in music therapy.

All parents said that they can imagine singing for their child after they have been discharged and 75% of parents can also imagine learning to play the sansula (Figure 3).

## 4. Discussion

This study shows that parents of premature infants perceive family-centered music therapy positively in terms of their own well-being and that of their child. The relaxing effect during the music therapy intervention played the most important role for the parents. The music therapy support was perceived by all parents as a positive change and enrichment during their hospital stay.

To promote bonding, relaxation is necessary [16,17], because the parents’ access to their child can be made more difficult by increased stress. This can prevent parents from correctly interpreting their child’s signals and respond adequately. Due to the concern for their own child, parents of preterm infants do not always succeed in relaxing, especially in the early days after birth [18,19]. Music therapy can (re)establish well-being and lead to relaxation [20]. 

A further important factor to build a bond between the parent and infant is the ability to interact. The more immature the premature infant, the lower his attention span and his ability to process stimuli [21]. In the highly stressful early phase after premature birth, music therapy can be an effective mean of communication to initiate the first dialogues and facilitate mutual relaxation, joy and playful exchange between parents and infants [22]. Feedback from parents in our study showed that music therapy helped about half of parents to come into contact with their child, thus promoting parent-child bonding [23].

Several studies have explored the effects of live music therapy on the mental well-being of preterm infants’ parents. Individually delivered live music therapy reduced anxiety, depression, and stress levels in mothers [24,25,26]. Combining live music therapy with kangaroo care lowered mothers’ anxiety more than kangaroo care alone [27]. Another report found higher short-term breast-feeding rates among mothers who had received music therapy compared to controls receiving standard care [28]. Of special interest is the fact that creative NICU music therapy reduced couples’ symptoms of anxiety and depression during the course of their NICU stay and beyond, while parents without music therapy experienced an increase of these symptoms [29]. Another study found that music therapy performed apart from the infants in a parent self-care group improved anxiety and stress levels [30]. Parents valued that they were able to relax and get distracted from their worries [30]. These studies show that live music therapy has benefits not only for preterm infants but also for parents. The effects were measurable on self-reported numeric rating scales, in open questionnaires, and by the state trait anxiety inventory. The results of our study complement this picture by adding evidence that family-centered music therapy promotes parents’ relaxation and parent-child interaction during their infant’s NICU stay.

Our study has several limitations. The parent survey was limited to the perceptions of the music therapy interventions, the reactions of the children and the socio-demographic characteristics of the parents in the therapy group. There was no assessment of the control group that received no music therapy and no validated tests measured parents’ stress or anxiety.

## 5. Conclusions

The family-centered music therapy was perceived by parents as a positive, supportive offer for themselves and for their children. The therapy promoted relaxation in parents and children as well as the parent-child interaction, which is necessary for the development of a stable bonding. Music therapy helps to relieve the families of prematurely born children in the difficult early days.

## Figures and Tables

**Table 1:** Clinical characteristics of the included patients in the therapy and the control group.

	Therapy Group (*n* = 40)	Control Group (*n* = 40)
Male, *n* (%)	22 (55%)	18 (45%)
Gestational age (weeks), mean ± SD (range)	28.6 ± 2.6 (23 + 6–31 + 5)	28.8 ± 2.5 (22 + 6–31 + 6)
Birth weight (g), mean ± SD (range)	1136 ± 404 (340–1790)	1147 ± 396 (360–2120)
Twins, *n* (%)	6 (15%)	2 (5%)
Died, *n* (%)	3 (8%)	2 (5%)

SD = standard deviation.

**Table 2:** Sociodemographic characteristics of the parents of the therapy group.

	*n* (%)
Parent	
Father	6 (19)
Mother	24 (75)
Father and mother together	2 (6)
Age (years)	
<25	3 (9)
26 to 30	4 (13)
31 to 40	24 (75)
>40	1 (3)
Education	
High school degree (13 years of education completed)	21 (66)
Degree “Realschule” (9 years of education completed)	5 (16)
Degree “Hauptschule” (8 years of education completed)	3 (9)
Other degree	1 (3)
No degree	2 (6)
Current professional situation (multiple answers possible)	
Parental time	25 (78)
Studying University/College	4 (13)
Professional working	7 (22)
Housewife/Houseman	3 (9)
Native language	
German	22 (69)
Other languages	10 (31)

## Data Availability

Original data will be made available to any qualified researcher upon request.
